# Societal actors’ perceptions of intersectoral collaboration and the participation of older adults in the development of age-friendly environments

**DOI:** 10.1186/s12877-026-08407-5

**Published:** 2026-10-06

**Authors:** Anna Nivestam, Madelene Törnblom, Maria Haak, Albert Westergren

**Affiliations:** 1 Faculty of Health Sciences, Department of Nursing and Health Sciences, Kristianstad University, Kristianstad, SE-291 88 Sweden; 2 Department for Research, Development and Education, Management for Psychiatry and Habilitation, Region Skåne, Lund, SE-223 63 Sweden

**Keywords:** Age-friendly environments, Collaboration, Healthy ageing, Inclusion, Participation, Preventive home visit

## Abstract

**Background:**

Older adults’ participation and intersectoral collaboration are central to the development of age-friendly environments. While previous research has emphasised the importance of these principles, less is known about how they are understood and enacted by societal actors in practice. This creates a need to explore societal actors’ perceptions of both intersectoral collaboration and older adults’ participation in the development of age-friendly environments.

**Method:**

The study had a participatory qualitative design. Data were collected through a World Café involving 19 societal actors in a Swedish municipal context. Participants represented several societal sectors relevant to age-friendly environments, including welfare, care, public health, governance, social sustainability, urban planning,mobility, civil society, and voluntary engagement.

**Findings:**

The findings showed that participants perceived intersectoral collaboration as requiring shared societal responsibility across multiple sectors, while responsibility risked becoming unclear when no actor had an explicit mandate to coordinate, prioritise and act. Intersectoral collaboration was viewed as a way of sharing resources, yet also as vulnerable when it relied mainly on personal relationships and informal networks rather than organisationally embedded structures. Similarly, older adults’ participation was highly valued when developing age-friendly initiatives, but participants questioned whether existing participation channels adequately reflect the diversity of older adults’ experiences and needs.

**Conclusion:**

Age-friendly work requires shared responsibility across societal actors, supported by clear coordination. Personal relationships and informal networks should be recognised as valuable for enabling collaboration, but they need to be complemented by formal structures to ensure sustainability over time. Moreover, greater efforts to reach seldom-heard older adults are needed. Doing so may contribute to more nuanced and age-friendly initiatives that better reflect the diverse needs of the older population.

## Background

Age-friendly environments aim to promote healthy ageing by adapting social and physical environments as well as municipal services to support older adults’ well-being across the life-course [1]. The age-friendly cities approach can be traced back to the early 1990s, when the United Nations (UN) Member States emphasised participation as a core principle in work related to ageing societies [2]. In 2002, the global challenge of “building a society for all ages” was further discussed at the Second UN World Assembly on Ageing in Madrid, where it was recognised that this work cannot be carried out by a single actor alone [3]. The World Health Organization (WHO) later developed the age-friendly cities framework [4], and in 2007, introduced eight core domains for an age-friendly city: outdoor spaces and buildings; transportation; housing; social participation; respect and social inclusion; civic participation and employment; communication and information; and community support and health services. Societal actors from all these domains were identified as important in the development of age-friendly cities. The initial guide was largely structured around a checklist of features within these domains that cities could use to assess their age-friendliness [4].

The framework was subsequently expanded through the Global Network for Age-friendly Cities and Communities [5] launched in 2010, and later handbooks for policymakers and societal actors [1, 6–8]. In this later work, the emphasis shifted from checklist-based assessment towards action across domains. Older adults’ participation and intersectoral collaboration were outlined as core principles, alongside multi-level governance and attention to inequality [1]. The terminology also broadened from age-friendly cities to the more inclusive concept of age-friendly environments. Thus, since the early 2000s, the UN and WHO have increasingly recognised that age-friendly work needs to be carried out both across sectors and with older adults themselves.

Intersectoral collaboration, defined as “cultivation and maintenance of partnerships with multiple societal actors”, is widely recognised as essential [7]. However, research shows that it is often challenging to achieve intersectoral collaboration in practice, due to competing priorities, limited resources (e.g. time and personnel), and lack of organisational structures [32]. Strategies such as establishing shared goals, providing leadership and forming collaborative forums have been proposed to support intersectoral collaboration [9], as well as place-based work and co-production by including older adults in the work [10]. Collaboration has been acknowledged as difficult but necessary in the development of age-friendly environments [11]. However, there remains a need to better understand how societal actors perceive the practical process of working together across sectors.

Moreover, since the beginning of the 1990s, older adults’ participation [2] has been recognised as important, but it remains unclear to what extent this principle is fulfilled in practice. According to the WHO toolkit for policymakers [7], older adults should be actively and genuinely involved throughout the entire process, from defining relevant issues to contributing to decision-making and implementation. Despite this, research highlights ongoing challenges in achieving meaningful participation, with concerns that involvement may at times remain symbolic or tokenistic rather than genuinely influential [12, 13]. Various approaches to engaging older adults have been described, including citizen juries, citizen panels, community juries, policy cafés [14], walking interviews, photovoice, and participatory mapping [15]. However, there is an ongoing discussion in the literature about what constitutes meaningful participation and whether participation risks becoming tokenistic [12, 13, 16].

Taken together, previous research has shown that age-friendly environments require both intersectoral collaboration and older adults’ participation [17]. However, less is known about how these principles are understood and translated into practice by the societal actors involved in age-friendly work. There is a need for a better understanding of how older adults’ perspectives are integrated into this work and how collaboration across sectors is perceived. Therefore, this study aimed to explore societal actors’ perceptions of intersectoral collaboration and older adults’ participation in the development of age-friendly environments.

## Methods

### Design

This study used a participatory qualitative design, with data collected through the World Café method [18, 19]. The method was chosen because the study aimed to explore societal actors’ perceptions of intersectoral collaboration as well as older adults’ participation in the work on age-friendly environments. This aim required a method that could generate collective reflection across organisational and sectoral boundaries, rather than only individual accounts. The World Café was therefore considered appropriate because it enables participants from different backgrounds to share experiences, compare perspectives, build on each other’s ideas and engage in discussion.

A method based on dialogue, successive rounds of discussion and collective reflection aligned well with the type of knowledge the study sought: not simply what individual actors thought, but how different societal actors collectively made sense of collaboration and older adults’ participation. The participatory nature of the study lay in the involvement of participants as knowledge contributors during the workshop, where they generated examples, discussed barriers and possibilities, documented key points, and contributed to a shared reflection in a plenary session.

Moreover, the workshop topics and questions were developed by the research team, informed by previous research on preventive home visits, older adults’ participation, and age-friendly environments. A municipal contact person provided contextual input and assisted with identifying relevant actors for recruitment. One older adult contributed to the study design, development of the workshop questions, and data collection during the World Café.

### Study context

The study was conducted in Sweden, in collaboration with a municipality in southern Sweden, which had a population of 85,908 inhabitants in 2025 [20]. Older adults constitute a substantial proportion of the population, with 23% being aged 65 years or older, which exceeds the national average of 21% [21]. Swedish municipalities have extensive responsibilities for services and environments that are relevant to older adults’ everyday lives, including social care, support for older adults, urban planning, housing-related issues, local infrastructure, social sustainability and collaboration with civil society. This makes the municipal level an important context for studying intersectoral work related to age-friendly environments. At the same time, many determinants of age-friendly environments extend beyond the responsibility of any single municipal department or organisation; therefore, participants outside the municipal organisation were also recruited.

The local setting was selected because the municipality had ongoing work related to age-friendly environments and because a local, in-person World Café made it possible to bring together actors from different sectors who influence older adults’ everyday environments. The study context therefore provided an opportunity to explore how societal actors in a real-world municipal setting perceived the intersectoral collaboration and older adults’ participation.

### Participant recruitment and sample

Purposive sampling [22] was used to identify participants who could contribute perspectives from different sectors relevant to age-friendly environments. Recruitment aimed to include actors representing as many domains as possible from the WHO framework for age-friendly environments: outdoor environments; transport and mobility; housing; social participation; social inclusion and non-discrimination; civic engagement and employment; communication and information; and community and health services [1].

A contact person from the municipality assisted by identifying individuals involved in the municipality’s steering and working groups for age-friendly environments and by providing contact details for potential participants. Additional potential participants were identified through organisational websites. The first author contacted potential participants by phone and e-mail and provided written information about the study.

In total, 48 people or organisations were invited, of whom 22 accepted the invitation. Three participants later withdrew or did not attend, resulting in a final sample of 19 participants. The sample included participants representing welfare, care and public health; education, children’s and young people’s development and educational leadership; management, governance and organisational development; democracy, working life and interest representation; social sustainability, urban planning and mobility; and civil society, voluntary engagement and relational support. These areas corresponded to several age-friendly domains.

The participants included both professional stakeholders and representatives from organisations or contexts where older adults were represented. Based on the background survey, four participants were aged 65 years or older (see Table 1). However, the study did not systematically classify participants according to whether they participated as older adults, professional stakeholders, civil society representatives or interest representatives. Therefore, the findings are reported as perspectives generated among participating societal actors. The sample characteristics are presented in Table 1.

**Table 1:** Sample characteristics

Total sample, *n*	*n* = 19
**Gender**, n	
Women	16
Men	3
**Age**, years, median (range)	53 (29–78)
**Highest education**, n	
Secondary education	3
Bachelor’s degree	8
Master’s degree	7
Doctoral degree	1

### Data collection

The World Café was conducted on 27 March 2026 at the university. A large lecture hall was used for plenary parts of the workshop, and six smaller rooms were used for group discussions. The event began with breakfast and a 30-minute mingling period, during which participants completed a background survey covering age, gender, education and work experience, and provided written informed consent.

The first author introduced the workshop by briefly presenting the WHO definition of healthy ageing and age-friendly environments [23], and by giving examples of the preventive home visit as one way of gathering older adults’ perspectives [24, 25]. The first author also explained the purpose of the workshop, the structure of the World Café and the practical procedures for the discussions.

Participants were pre-assigned to groups and received a schedule indicating times and rooms. In total, six discussion groups were held, and three questions were discussed. In two rooms, the same question was discussed simultaneously. The questions were: “How do you currently gather the perspectives of older adults — and how are these perspectives used in your organisations to create age-friendly environments?”, “How could the perspectives of older adults be used more in your work to create age-friendly environments?”, and “How can you collaborate to use the perspectives of older adults and create age-friendly environments?”

In four rooms, a designated host took notes and summarised the previous group’s discussion for incoming participants. The host’s role was to support continuity between discussion rounds, not to moderate or direct the content of the discussion. The room hosts included the second and the fourth authors, a master’s student, and an older adult who had contributed to the study design. Discussions began with brief introductions and one minute of silent reflection, followed by group discussion. Participants documented key ideas on Post-it notes or paper. The room host took notes during all discussions. Each discussion lasted 25 min. At the end of each round, participants wrote final reflections and then rotated to a new room.

In the two rooms without a host, participants followed the same structure, but discussion took place without any host taking notes or summarising what had been said. In these rooms, the first author ensured that the procedure was understood and that groups moved between rooms according to the schedule, but did not stay in the room during the discussions. Group size varied from two to five participants. After all the discussion rounds were complete, participants reconvened in the lecture hall for a plenary reflection. During this session, the hosts summarised the group discussions, and participants were invited to add comments, clarify points and contribute to a shared understanding of the issues raised.

The empirical material consisted of audio recordings of group discussions, host notes, Post-it notes written by the participants, and notes from the plenary reflection taken by the second author. After the World Café, the first author transcribed two discussions with assistance from the AI software Scribe. Three recordings were professionally transcribed. In one case, a recording failure occurred; this was compensated for by using the host’s detailed written notes from that discussion.

### Data analysis

Data were analysed using reflexive thematic analysis, informed by Braun and Clarke [26]. This approach was appropriate because the study aimed to interpret patterns of meaning in collectively generated qualitative material. The analysis was conducted as an iterative and interpretive process, moving from familiarisation with the material, to coding, theme development and refinement, and finally to analytical interpretation in relation to the study aim.

First, the first author familiarised herself with the material by reading the transcripts and written notes several times, listening to the audio recordings and writing initial reflections. The second author listened to one group discussion and made notes on aspects relevant to the study aim. The first and second authors then discussed their initial impressions to reflect on emerging meanings and ensure that the analysis remained grounded in the material.

All material, including transcripts, host notes, and Post-it notes, was imported into NVivo 14. The first author conducted the initial coding. Codes were assigned to segments of data that captured relevant meanings in relation to the aim, such as responsibility, collaboration, communication, resources, participation, digitalisation, and exclusion. Coding was inductive in the sense that codes were developed from the material, but it was also informed by the study aim.

In the next step, the first author reviewed the codes and grouped codes with related meanings into preliminary sub-themes. These sub-themes were discussed with the second and fourth authors, who provided reflections on their relevance, coherence and relation to the empirical material. Through this process, the analysis moved from descriptive groupings of codes to broader patterns. Preliminary findings were presented to participants at an online seminar. Participants were invited to comment on whether they recognised the findings and whether important aspects were missing. Participants recognised the results and did not identify missing aspects.

As the analysis developed further, the research team refined these areas into more interpretive themes. This led to the development of themes concerning shared but unclear responsibility across sectors, the tension between relationship-based and structurally embedded collaboration, uneven accessibility of participation, resource sharing, and exclusion. These themes were discussed among all co-authors and refined to ensure that they were internally coherent, distinct from each other and grounded in the empirical material. The final themes were then further refined and written up by the first author, with input from all co-authors.

### Reflexivity

The research team’s previous experience and roles may have influenced both data generation and interpretation. The first author has previous research experience in preventive home visits, older adults’ participation, and the use of older adults’ perspectives at a societal level. This background informed the development of the study, the workshop questions and the introductory presentation. By introducing examples related to preventive home visits and age-friendly environments, the first author may have foregrounded certain issues, such as participation, collaboration and the use of older adults’ perspectives.

During the World Café, the first author coordinated the workshop and ensured that the correct procedure was followed. Room hosts supported the process by taking notes and summarising previous discussions for incoming participants. These roles may have influenced the discussions by shaping how continuity between rounds was maintained and which points were highlighted in summaries. At the same time, participants were encouraged to steer the content of the discussions themselves, generate their own examples, document key points and add reflections during the plenary session.

In the analysis, the researchers’ prior knowledge may have influenced the interpretation of patterns in the data. The first, third, and fourth authors had previous knowledge of age-friendly environments, while all authors had experience conducting preventive home visits. To strengthen reflexivity, preliminary interpretations were discussed among co-authors, and the findings were presented to participants for recognition and comment. The analysis should therefore be understood as an interpretive account developed through engagement between the participants, the empirical material, the researchers’ prior knowledge and reflexive discussions within the research team.

## Findings

The findings are presented in relation to the two parts of the study aim: societal actors’ perceptions of intersectoral collaboration, and older adults’ participation in the development of age-friendly environments. However, intersectoral collaboration may include collaboration with older adults, and some societal actors may themselves be older adults or represent organisations in which older adults’ perspectives are central. The distinction between societal actors and older adults should therefore not be understood as fixed, as the two groups may partly overlap in practice.

Intersectoral collaboration and older adults’ participation were valued as central principles, but the findings show that these principles are not self-sustaining. Responsibility, coordination, as well as both informal relationships and formal structures are required for intersectoral collaboration. Resources (e.g., competencies and personnel) can be shared among actors, enabling more efficient age-friendly work. Older adults’ participation needs deliberate efforts to reach beyond the most visible voices. The following themes therefore point to a common underlying issue: age-friendly work requires not only shared commitment, but also organisational and participatory conditions that make this commitment actionable, sustainable and inclusive.

### Taking responsibility across sectors, but unclear ownership

Participants described responsibility as a central condition for working with intersectoral collaboration when creating age-friendly environments, but they also suggested that responsibility was often dispersed and unevenly recognised across organisations. In the discussions, they all agreed that age-friendly work concerns many different parts of society, including care and welfare services, public health, urban planning, transport, housing, civil society and voluntary organisations. From this perspective, they discussed how responsibility could not be limited to organisations that have older adults as their primary target group. Instead, participants perceived age-friendly environments as requiring broader societal ownership. This is shown in the following transcripts from the World Café:

“…and we already talked about this in the other groups [during the World Café], …that we cannot do this on our own, and as you also said, really….”

“Yes, you do not work specifically with older people, but still….”

“No, not really at all.”

“…you have quite a lot that you can contribute.”

At the same time, the discussions indicated an important tension. When older adults are only one of several target groups, the societal actors may not perceive age-friendly work as their main responsibility. This is shown in the following transcripts from the World Café:

“…because we don’t really work specifically with older people to any great extent.”

“No.”

But they are included in our thinking when we work in different ways… of course.

“In what ways?”

“Well, for example, in relation to the wishes people have. I mean, we don’t have a specific focus on older people, but on everyone.”

This tension was particularly evident in discussions about actors whose core tasks are not explicitly framed around ageing, such as technical departments, transport actors, businesses or organisations working with broader community development. Participants suggested that these organisations may still shape older adults’ everyday lives, but that their responsibility for working with age-friendly environments in particular remains implicit rather than formally acknowledged.

This ambiguity around responsibility was not only described as a practical challenge, but also as a governance issue. Participants suggested that age-friendly work requires both distributed responsibility and some form of coordination. Without a shared understanding of who is responsible for what, the work with age-friendly environments risks being treated as an optional addition rather than as an integrated part of organisational planning and decision-making. Thus, the discussions highlighted that the issue was not simply whether different actors were willing to collaborate, but whether there were organisational conditions that made responsibility visible, legitimate and actionable. This is shown in the following Post-it note from a discussion during the World Café:

“Not all actors within the municipality are aware of their responsibilities.”

### Balancing relationship-based collaboration with structural anchoring

Discussions highlighted the need for both informal, relationship-based collaboration and more structurally embedded forms of collaboration. Participants described communication and networking as central processes for enabling intersectoral collaboration. This is shown in the following Post-it notes from a discussion during the World Café:

“Relationships and communication are important”, “It tends to become person-dependent.”

The participants repeatedly emphasised the importance of knowing whom to contact, being able to invite others into dialogue, and maintaining networks over time. Collaboration was therefore described as dependent on personal relationships, mutual trust and the continuous “cultivation” of contacts.

However, the discussions also revealed that this relational basis for collaboration was perceived as fragile. Participants emphasised that collaboration depends mainly on specific individuals; it may become vulnerable to staff turnover, changing priorities or uneven engagement between organisations. They suggested that collaboration needs to be supported by more stable structures, such as recurring forums, formal agreements, designated contact persons, shared platforms, policies or leadership support. In this sense, participants did not describe informal relationship-based collaboration and structural collaboration as opposing alternatives, but as mutually dependent conditions. Relationships helped initiate and sustain collaboration, while structures were perceived as necessary to make collaboration less person-dependent and more durable.

Participants proposed an idea of a shared platform which illustrates this tension. They suggested that such a platform could make ongoing activities visible, enable the sharing of examples and experiences, and help organisations ask questions or identify relevant partners. This is shown in the following transcripts from the World Café:

“…it might perhaps have been simple to have a platform somewhere where people could add the things we offer.”

“Yes.”

Where all actors could add information, saying: this is what we have right now.

“Yes.”

“We are looking for someone who could provide input on this, or we are going to start this… yes, I don’t know.”

“But I’m thinking of something that we could all have as a shared forum.”

“Yes, and perhaps also some way of asking questions about whether this is something I could refer someone to, that is….”

“Yes, definitely.”

“Some way in, so to speak, where one could discuss things further.”

“Exactly, that there is someone in each organisation who keeps track of things.”

“What it looks like — a kind of local monitoring, in a way.”

“Mmm.”

“That is probably something one sometimes misses….”

At the same time, they noted that a platform would only be useful if responsibility for maintaining it was clearly assigned, for example through designated contact persons in each organisation. The discussions indicate that structural solutions were seen as requiring both technical infrastructure and organisational commitment. Similarly, the participants suggested a “navigator”, someone who could help actors find the right contact point within complex organisational systems, which reflected the perceived complexity of intersectoral work. This suggestion implies that participants recognised that collaboration is not only a matter of willingness, but also of organisational accessibility.

### Sharing resources across sectors to enable more efficient age-friendly work

Participants discussed resource sharing across sector boundaries as a possibility arising from intersectoral collaboration. They described how different actors could contribute different forms of expertise, personnel, services, spaces or knowledge. For example, participants suggested that care personnel should be able to focus on care, while other actors may be better placed to provide social, religious, cultural, and voluntary activities. They also discussed collaboration between care services, housing, transport, outdoor environments and health care as a way to combine different areas of expertise. One participant described how involving a service team in care units enabled care personnel to focus more on their core duties:

This is shown in the following transcripts from the World Café:

“ … It works really well [the care staff], have more time [for other duties].”

Moreover, participants suggested that sharing resources, exchanging experiences and learning from good examples may reduce duplication and help organisations avoid developing solutions from scratch. They perceived intersectoral collaboration as having the potential to contribute to a more efficient use of resources, provided that there are structures for coordination, communication and follow-up. Participants’ discussions show that resource efficiency was a valued possibility and a rationale for collaboration.

**The paper will now consider the second aspect of the aim of this study: **to describe participants’ perceptions of older adults’ participation in the development of age-friendly environments.

### Valuing older adults’ participation while questioning whose voices are heard

Participants described the gathering of older adults’ perspectives as fundamental to age-friendly work. They emphasised that older adults’ experiences, needs and preferences should inform the development of age-friendly environments. The discussions identified several existing ways of gathering such perspectives, including surveys, resident councils, pensioners’ councils, focus groups, complaint systems, interviews, home visits, follow-up calls, care encounters, meetings, seminars and training activities. Participants also mentioned that employing older adults within organisations could be one way of integrating older adults’ perspectives into everyday practice.

However, participants reflected on whose perspectives are actually represented through these methods. They questioned whether current participation practices capture the diversity of older adults’ experiences and needs. They raised concerns that the same older adults may be repeatedly invited to participate through established forums such as associations, pensioners’ organisations, councils, boards and local political structures. This is shown in the following transcripts and Post-it notes from the World Café:

“Those who are most often heard are those who are active and most inclined to complain.”, “…we have…the pensioners’ council. And many pensioners’ associations that have an influence politically. But that group of older adults who are not active in any association, how do we include their perspective?”

The participants discussed the risk that these participation processes may lead to inequalities. Those who are already active, confident and familiar with formal participation channels may have repeated opportunities to influence age-friendly work, while seldom-heard older adults (groups that participants perceived as less likely to be involved through traditional forms of participation) may have fewer opportunities to contribute. Participants therefore did not present participation as automatically inclusive. Rather, they perceived inclusion as a condition that requires deliberate outreach, methodological variation and attention to barriers.

Digitalisation was discussed as a particularly ambivalent condition. Some participants saw digital tools, including websites and online surveys, as potentially useful for collecting input and managing feedback. At the same time, they emphasised that digital tools may exclude older adults who lack digital skills, devices, internet access or confidence in using digital services. Digital participation was not understood as neutral by the participants; it was perceived as both a possible resource and a possible mechanism of exclusion. This is shown in the following transcripts from the World Café:

“…it is easy to think that this digital aspect, that… everyone will become digital in the future, but that is not the case.”

“No.”

“Because one might think that… the more people work with digital tools in working life and so on, that this will lead to it, but in the long term there will still be an age-related aspect.”

“Yes.”

When… cognitive abilities nevertheless decline, and it becomes more difficult to learn new things.”

“Yes.”

“So we will always have this non-digital group.”

“Even if, over time, it may become somewhat smaller, it will always exist.”

“Yes.”

“So it is still an important nut to crack.”

### Making age-friendly initiatives responsive, but not always representative

Participants perceived that older adults’ participation may support the development of age-friendly initiatives based on older adults’ needs. Examples discussed by participants included adapting working methods in care homes in response to older adults’ wishes, conducting in-depth interviews about accessibility for people with hearing impairment, and linking domains such as transport, housing and home care to better address older adults’ everyday needs. These examples indicate that participants could identify situations where older adults’ perspectives had informed organisational reflection or practical adjustments. Participants perceived that when older adults’ perspectives are systematically gathered, shared and acted upon, age-friendly work may become more responsive to older adults’ needs. At the same time, participants identified a risk that age-friendly initiatives may not address the diversity of older adults’ needs if they are shaped primarily by those already visible in existing participation structures.

Participants also discussed the under-representation of older adults with a foreign background in established associations. One participant reflected that using the same approach to reach everyone may not be sufficient:

This is shown in the following transcripts from a discussion during the World Café:“

“…Often, I think it is not really a lack of willingness. Rather…we reach out to everyone in the same way. But for that target group, one needs to think differently… it is difficult.”

## Discussion

This study explored societal actors’ perceptions of intersectoral collaboration and older adults’ participation in the development of age-friendly environments. The findings showed that intersectoral collaboration requires shared societal responsibility that extends across multiple sectors, but it is also a responsibility that risks becoming unclear when no actor has an explicit mandate to coordinate, prioritise and act. Moreover, intersectoral collaboration was viewed as a way of sharing resources, yet also as vulnerable when it relied mainly on personal relationships and informal networks rather than organisationally embedded structures. Similarly, older adults’ participation was highly valued as a way of developing age-friendly initiatives, but participants questioned whether existing participation channels reflect the diversity of older adults’ experiences and needs.

The findings showed that creating age-friendly environments through intersectoral collaboration requires shared responsibility across societal actors. However, when responsibility is distributed among many actors, it may remain unclear who has the mandate to coordinate, prioritise and act. The participants’ discussions suggest that shared responsibility needs to be accompanied by clear coordination. Previous research highlights the importance of leadership [9], having persons committed to lead this work or a person (a champion) who really promotes the work and takes on the critical leadership [27]. Which societal actor should hold this responsibility is not clearly described in either international policy documents [1] or Swedish national policy documents [28]. In Sweden, municipalities have extensive responsibilities for older adults’ health and well-being, but there is no specific policy on age-friendly environments or on who is accountable for this work. Such policy guidance could make the responsibility clearer, including for actors who do not currently recognise their role in creating age-friendly environments. Previous research also highlights the need to embed age-friendly policies at all levels of municipal government, rather than treating them as the responsibility of a single department or sector [29]. Swedish national policy documents therefore need to be reviewed so that the issue of accountability is more clearly included in broader public health matters concerning older adults.

Another important contribution of the findings is that they showed how intersectoral collaboration depends on both relationship-based collaboration and more structurally embedded forms of collaboration. Participants described informal communication and networking as important for intersectoral collaboration, which is turn, could be fruitful for the creation of age-friendly environments. This corresponds with previous research showing that trust, relationships and cross-sector engagement are central to age-friendly community change [10, 11]. However, the present findings also showed that collaboration based primarily on personal networks was perceived as vulnerable. If intersectoral collaboration depends on specific individuals, it may be difficult to sustain when personnel change, priorities shift or engagement varies between organisations. Previous research has largely focused on the structural conditions needed to enable intersectoral collaboration [30]. However, the present findings suggest that, in practice, personal relationships and informal networks may have a significant influence on what is actually done. Participants in the present study suggested a need for more stable structures, such as recurring forums, shared platforms, designated contact persons, and leadership. This is in line with what is recommended in previous research [30]. This does not mean that informal relationships were seen as unimportant. Drawing attention to the literature about collective governance [31] (“… where one or more public agencies directly engage non-state stakeholders in a collective decision-making process that is formal, consensus-oriented, and deliberative and that aims to make or implement public policy or manage public programmes or assets.” p 544), highlights that the relationship between people is as important as structures for collaboration, to active successful outcomes. For example, engagement, mutuality, trust, and commitment are important aspects [31]. Personal networks can be understood as part of the relational and motivational processes that foster collaboration, whereas formalised networks can be understood as part of the structures that enable collective action. The present findings suggest that participants viewed relationships and structures as mutually dependent. Personal contacts may enable action, but structural anchoring may be needed to make collaboration durable and less dependent on individual commitment.

Regarding older adults’ participation in the development of age-friendly environments, participants emphasised that older adults’ perspectives should inform age-friendly work, but they also questioned whose perspectives are represented through existing participation processes. This distinction is important because participation is a central principle of age-friendly work [1, 32], but it does not automatically result in influence, representation or inclusion. The findings suggest that established participation channels may repeatedly involve the same older adults: those who are active, organised, digitally connected, or familiar with civic forums.

This finding connects to previous research on meaningful participation and tokenism in age-friendly and policy-related work [12, 13, 16]. Earlier studies have argued that participation should involve more than consultation and should enable older adults to influence decisions that affect their lives [12, 13]. However, even when older adults are invited to participate, established processes may reproduce exclusion if they primarily reach those who are already able and willing to engage in formal settings. Inclusive participation therefore requires active efforts to reach seldom-heard older adults, including those who are socially isolated, digitally excluded, cognitively or physically frail, or living in residential care settings. Approaches such as co-production, spatial methods [33] and preventive home visits [33–35]. may broaden participation, although no single approach will reach all groups. Therefore, additional efforts are needed to support the participation of older persons living in for example residential care settings [36].

### Strengths and limitations

A strength of this study is that it included societal actors representing several domains relevant to age-friendly environments. This enabled discussions across organisational and sectoral boundaries and made it possible to explore age-friendly work as a shared societal issue. The World Café method was suitable for generating collective reflection on a complex topic and allowed participants to build on each other’s experiences and ideas. The use of several types of material, including audio recordings, host notes, Post-it notes and plenary reflections, strengthened the empirical basis and made it possible to analyse both spoken discussions and collectively documented ideas.

The study also has limitations. Although 48 people or organisations were invited, the final sample consisted of 19 participants. The findings should therefore be interpreted in relation to the perspectives represented among those who participated, rather than as reflecting all relevant societal actors. The study also involved participants who were willing and able to attend a World Café on age-friendly environments, which may have resulted in an over-representation of actors who were already engaged in, interested in, or familiar with issues related to older adults’ participation and intersectoral collaboration. This may have influenced the emphasis placed on collaboration, participation and inclusion in the findings.

Another limitation concerns the World Café format. The method generates collectively developed ideas rather than detailed individual narratives. As a result, the material may be less rich in verbatim quotations than interviews or focus groups, and it may sometimes be difficult to distinguish individual participants’ views from collectively produced reflections. The findings should therefore be understood as collectively generated perspectives among participating societal actors. Moreover, the World Café method does not allow for an in-depth exploration of the phenomenon. Therefore, further research is needed to gain a deeper understanding of intersectoral collaboration and older adults’ participation.

## Conclusions

Age-friendly work requires shared responsibility across societal actors, but shared responsibility is not sufficient unless responsibilities are made visible, coordinated and organisationally anchored. The findings indicate that societal actors recognise their potential contributions to age-friendly environments, yet unclear ownership may limit the extent to which this work becomes an integrated part of planning, prioritisation and action. Personal relationships and informal networks should therefore be recognised as valuable conditions for initiating and enabling collaboration, but they need to be complemented by formal structures, designated contact points and recurring forums to make collaboration sustainable over time. Intersectoral collaboration may also support more efficient use of resources when knowledge, competences and services are shared across sectors. However, such collaboration requires structures that enable communication, follow-up and collective learning. Finally, older adults’ participation needs to extend beyond established channels that may primarily reach already active and visible groups. Greater efforts are needed to reach seldom-heard older adults through varied and accessible forms of participation. Such efforts may contribute to more responsive, inclusive and nuanced age-friendly initiatives that better reflect the diverse needs and everyday experiences of the older population.

## Data Availability

The datasets generated during and analysed during the current study are not publicly available for confidentiality reasons but are available from the corresponding author upon reasonable request.
